# Supplementary material for: RPA activates the XPF‐ERCC1 endonuclease to initiate processing of DNA interstrand crosslinks
Source: EMBO J. 2017 Jun 12;36(14):2047–60. doi: 10.15252/embj.201796664 (PMC5510000; doi:10.15252/embj.201796664)
Supplement: Supplementary file 1 — Appendix [file EMBJ-36-2047-s001.docx]

**Appendix – table of contents:**

Appendix Table S1 – sequences of DNA substrates. Page 2

Appendix Figure S1 – schematic representation of substrates. Page 3

Appendix Supplementary Methods: Substrate generation/synthesis Page 4

Appendix reference Page 5

| **Code** | **Sequence of DNA oligonucleotides** |
| --- | --- |
| 1 | 5’-ATAAATATTTTTTATTAATAATAGATCACCTTTCTTTCTCTTCTCCCCTT-3’ |
| 5 | 5’-TTCCCCTCCTCTCCTTCCTTCCTGATCTATTATTAATAAAAAATATTTAT-3’ |
| 6 | 5’-AAGGGGAGAAGAGAAAGAAAGG-3’ |
| 7 | 5’-AAGGGGAGAAGAGAAAGAAAG-3’ |
| 8 | 5’-AAGGGGAGAAGAGAAAGAAA-3’ |
| 9 | 5’-AAGGGGAGAAGAGAAAGAA-3’ |
| 10 | 5’-AAGGGGAGAAGAG-3’ |
| 11 | 5’-GGAAGGAAGGAGAGGAGGGGAA-3’ |
| 12 | 5’-(UUCCCCUCCUCUCCUUCCUUCC)TGATCTATTATTAATAAAAAATATTTAT-3’ |
| 13 | 5’-ATAAATATTTTTTATTAATAATAGATC**X**CCTTTCTTTCTCTTCTCCCCTT-3’ |
| 14 | 5’Bio-TTCCCCTCCTCTCCTTCCTTCC**Y**GATCTATTATTAATAAAAAATATTTAT-3’P |
| CL-1 | 5’ -ATAAATATTTTTTATTAATAATAGATC**X**CCTTTCTTTCTCTTCTCCCCTT-3’  3’P-TATTTATAAAAAATAATTATTATCTAG**Y**CCTTCCTTCCTCTCCTCCCCTT-5'Bio |

**Appendix Table S1.** **Sequence of DNA oligonucleotides used to generate the DNA substrates for this study. X** is (6-amino)- hex-1-yl]-8-amino-2'-deoxyadenosine labelled with Azidohexanoic acid NHS ester and Y is 5-(octa-1,7-diynyl)-2'-deoxyuridine; ‘Bio’ stands for 5’-biotin; ‘P’ stands for 3’-phosphate. Red lines represent a triazole interstrand crosslink (see Figure 4A); non-complementary regions are underlined; sequence in brackets stands for RNA sequence.


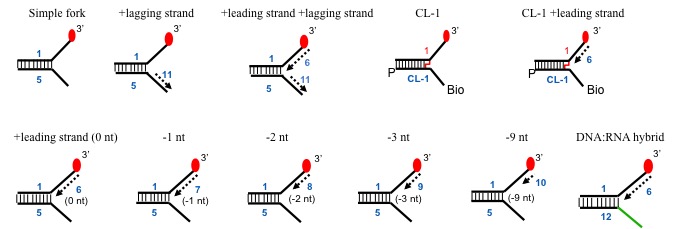


**Appendix Figure S1. A schematic representation of the DNA substrates utilised in this study.** The code for the DNA oligonucleotides (as stated in the table 1) used to assemble the DNA substrates are indicated in blue. ‘P’ stands for 3’-phosphate; ‘Bio’ stands for 5’-biotin; green lines represent regions of RNA; red number represent the location of the triazole ICL with respect to the ss/dsDNA junction.

**Supplementary Methods: Generation of triazole-containing crosslinked substrates**

Crosslinked oligonucleotides were generated in an analogous manner to published procedure (Kocalka, El-Sagheer et al., 2008). In brief, standard DNA phosphoramidites, solid supports (controlled pore glass) and additional reagents were purchased from Link Technologies, Glen research, and Applied Biosystems. All oligonucleotides were synthesized on an Applied Biosystems 394 automated DNA/ RNA synthesizer using a standard 1.0 *μ*mol phosphoramidite cycle of acid-catalyzed detritylation, coupling, capping, and iodine oxidation. Stepwise coupling efficiencies and overall yields were determined by the automated trityl cation conductivity monitoring facility and in all cases were >98.0%. All β-cyanoethyl phosphoramidite monomers were dissolved in anhydrous acetonitrile to a concentration of 0.1 M immediately prior to use. The coupling time for normal A, G, C, and T monomers was 60 s and the coupling time for 5'-(4,4'-Dimethoxytrityl)-N6-benzoyl-N8-[6-(trifluoroacetylamino)- hex-1-yl]-8-amino-2'-deoxyadenosine, 3'-[(2-cyanoethyl)- (N,N-diisopropyl)]-phosphoramidite (amino modified C6 dA) (Link) and 5'-Dimethoxytrityl-5-(octa-1,7-diynyl)-2'-deoxyuridine, 3'-[(2-cyanoethyl)-(N,N-diisopropyl)]-phosphoramidite (alkyne) (Glen research) was extended to 840 s. Oligonucleotides were cleaved from the solid support and deprotected by exposure to concentrated aqueous ammonia solution for 60 min at room temperature followed by heating in a sealed tube for 5 h at 55°C. Oligonucleotides were purified by HPLC using triethyl ammonium bicarbonate (TEAB) buffer.

The amino-modified oligonucleotide was labelled with azidohexanoic acid NHS ester to form the azide oligonucleotide (13 in table 1). In brief, 6-Azidohexanoic acid NHS ester (1 mg) was added in DMSO (80 µL) post-synthetically to the freeze-dried amino-modified oligonucleotide (oligonucleotide modified with amino C6 dA) (200 nmole) in 0.5 M Na_2_CO_3_/NaHCO_3_ buffer, pH 8.75 (80 µL). After 4 h at room temperature the fully-labelled oligonucleotide was purified by reversed-phase HPLC.

Azide and alkyne containing strands were dissolved together in 0.2 M NaCl (110 µL, 50 µ M) and were annealed. A solution of Cu^I^ catalyst was prepared by dissolving tris-hydroxypropyltriazole ligand (THPTA) (0.5 mg), sodium ascorbate (0.2 mg) and CuSO_4_.5H_2_O (0.03 mg) in water (15 µL), and degassing with argon. The catalyst solution was immediately added to freshly annealed DNA strands and incubated at room temperature for 2 hours under argon prior to desalting. Reactions were desalted using NAP-25 (GE Healthcare) followed by purification using 8 % PAGE Urea gels. Gel regions containing cross-linked material were excised and DNA material recovered by the crush and soak method. In brief gel samples were crushed to a powder and soaked in water (30 mL) for 18 hours at 37°C with vigorous shaking. Samples were filtered and the resulting solution desalted using NAP-25 (GE Healthcare). The pure cross-linked oligonucleotides were characterised by mass spectrometry and lyophilised prior to use.

**Reference**

Kocalka P, El-Sagheer AH, Brown T (2008) Rapid and efficient DNA strand cross-linking by click chemistry. Chembiochem 9: 1280-5
