# Supplementary material for: RPA activates the XPF‐ERCC1 endonuclease to initiate processing of DNA interstrand crosslinks
Source: EMBO J. 2017 Jun 12;36(14):2047–60. doi: 10.15252/embj.201796664 (PMC5510000; doi:10.15252/embj.201796664)
Supplement: Supplementary file 2 — Expanded View Figures PDF [file EMBJ-36-2047-s002.pdf]

Expanded View Figures

Figure EV1. Purification and validation of nuclease activity of human recombinant XPF-ERCC1 (XE).

A Purified XPF-ERCC1 (XE) analysed on an SDS–PAGE gel (4–12%) stained with InstantBlue (left-hand panel) and Western blot analysis (right-hand panel). WT = wild type XE; D676A = XE mutated to substitute aspartic acid residue 676 of XPF with alanine.

B Nuclease activity of WT and D676A forms of XE on a "simple fork" substrate. The substitution of metal-binding residue in XPF (D676A) renders the XE complex devoid of any nuclease activity. Red circles denote 3′[^{32}P]-radiolabelled nucleotides.

C (Top panel) Nuclease activity of XE on a "simple fork" substrate over a time course. (Bottom panel) Quantification of intact substrate and incision products expressed as a percentage of initial substrate as in top panel, *n* = 2.

© 2017 The Authors

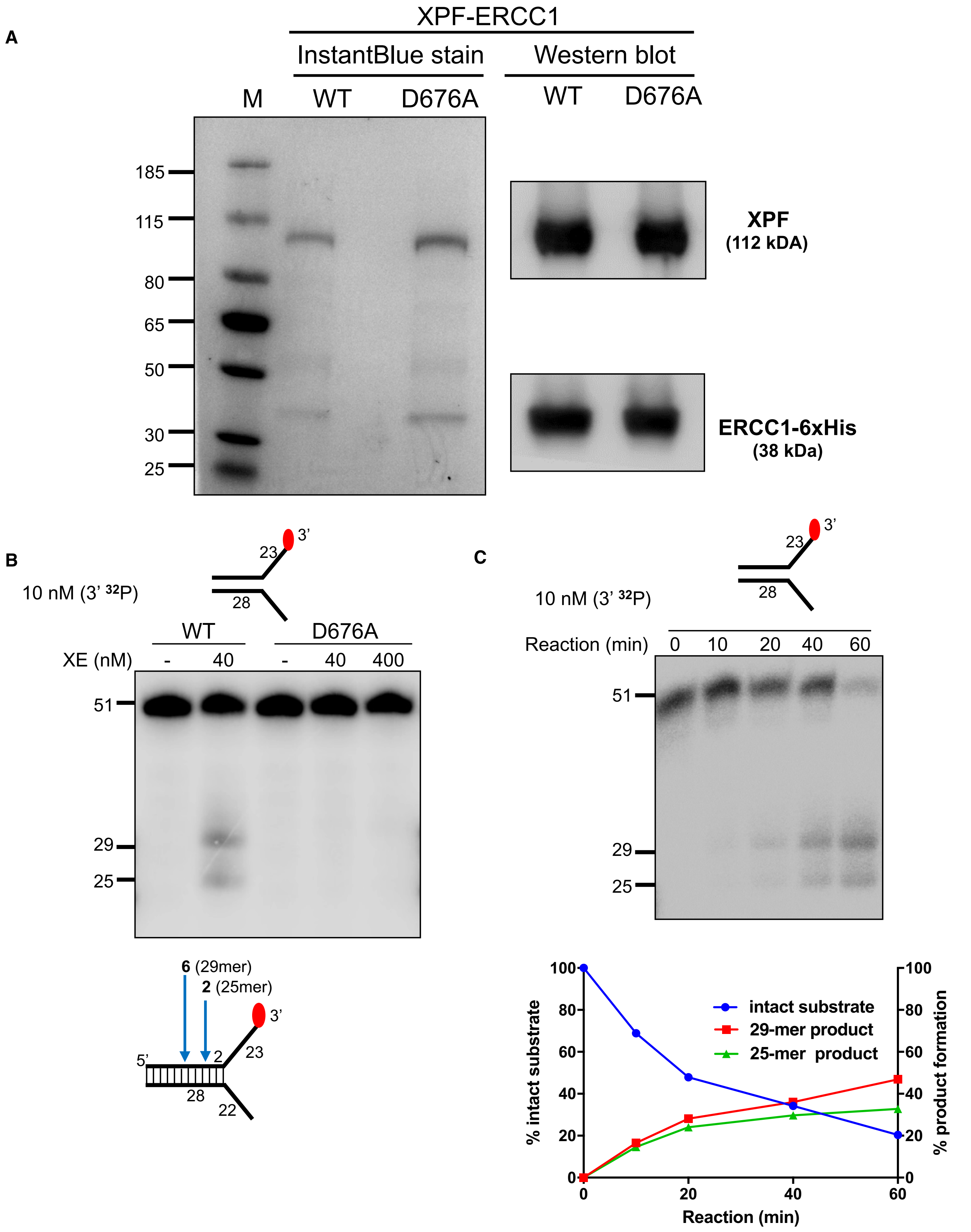

Figure EV1.

 © 2017 The Authors

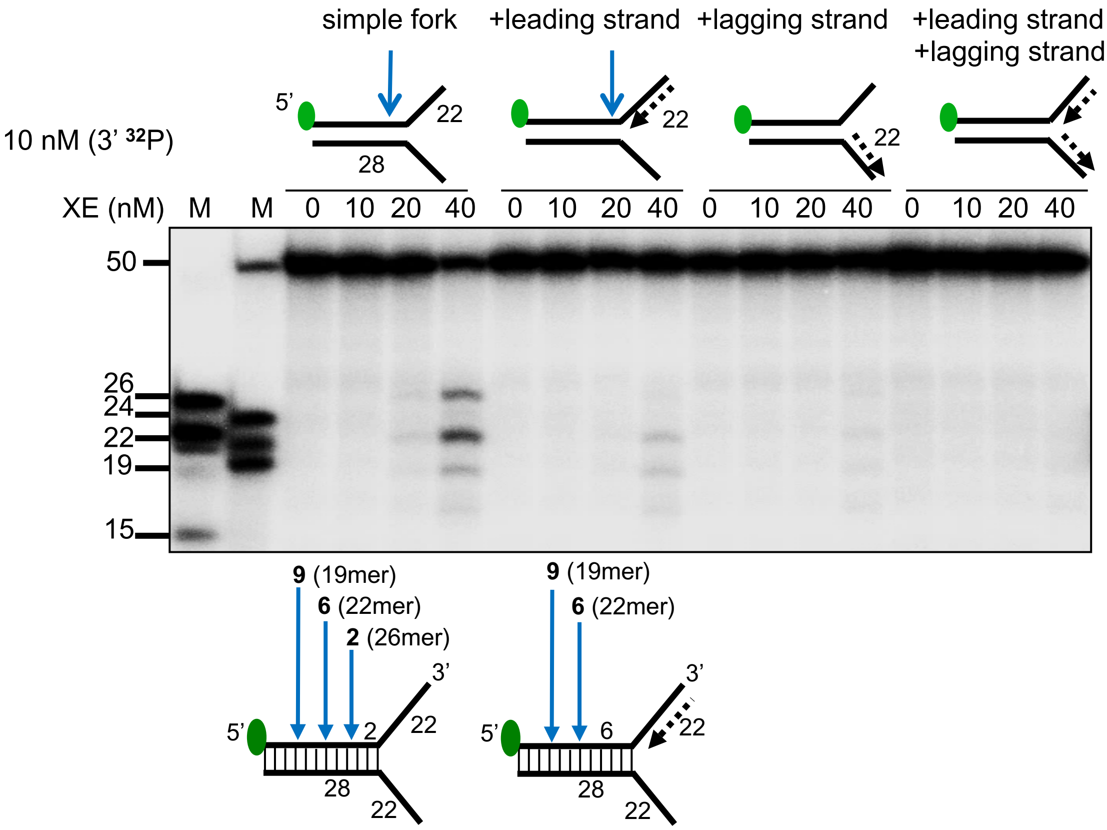

Figure EV2. 5′-radiolabelling of the DNA substrates reveals additional minor incisions by XPF-ERCC1, within the duplex region.
(Top panel) Nuclease activity of XE on the indicated fork substrates. Green circles denote 5′[^{32}P]-radiolabelled nucleotides. (Bottom panel) A schematic representation of the position of XE incisions: the positions of incision relative to the fork junction are indicated in bold and the size of 5′[^{32}P]-labelled incision products is indicated in parentheses.

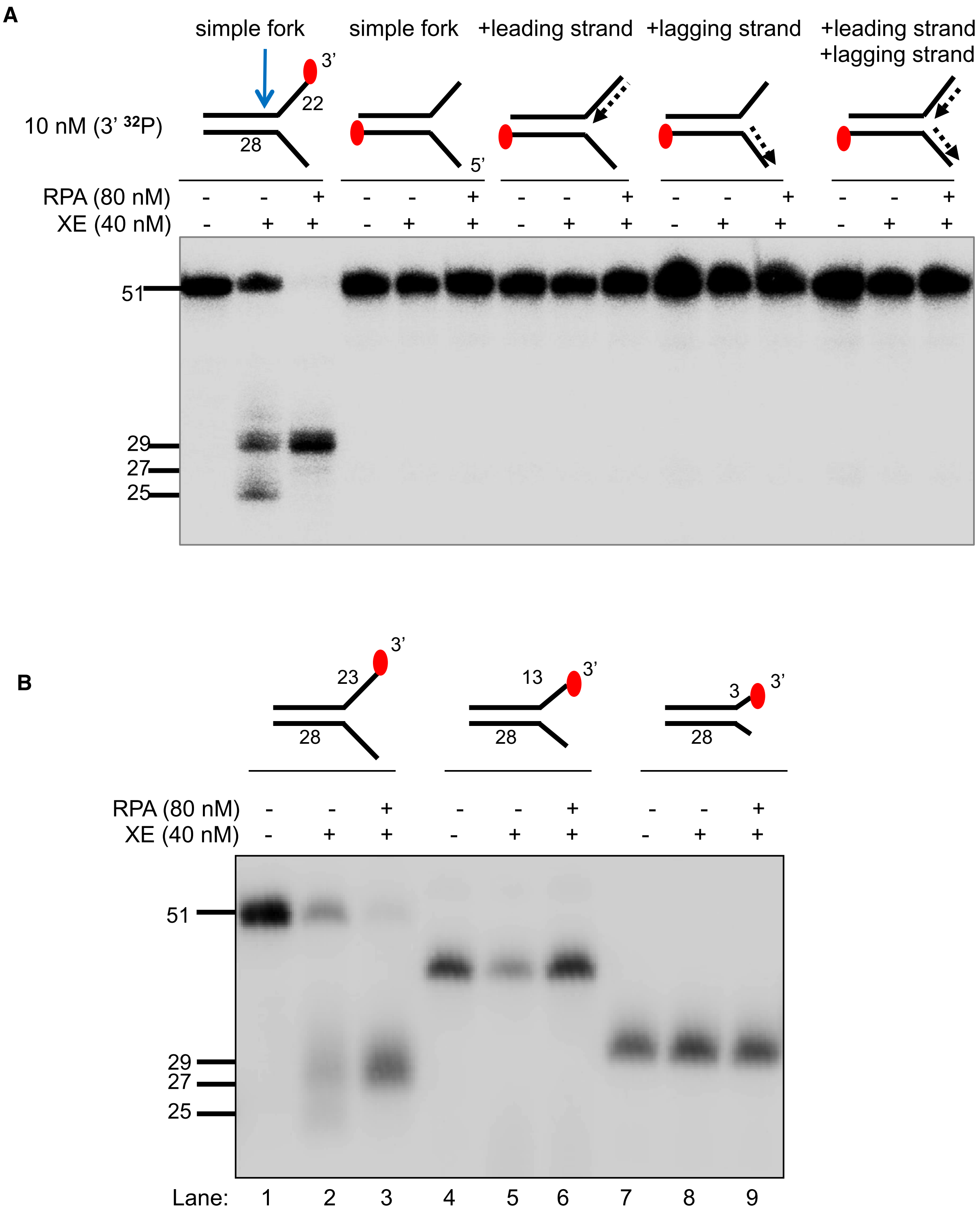

Figure EV3. RPA specifically stimulates XPF-ERCC1 activity on the strand with a 3′-ssDNA arm of a fork structure and its stimulation requires a minimum of 23 nucleotides ssDNA arms.

A The nuclease activity of XE in the presence and absence of RPA on fork substrates 3′[^{32}P]-radiolabelled either on the "top" or the "bottom" strand. XPF-ERCC1 activity is not detectable on the bottom strand of the fork substrates, which have a 5′-ssDNA arm.

B The nuclease activity of XE in the presence and absence of RPA on simple fork substrates containing arms of 23, 13 or 3 nucleotides.

Source data are available online for this figure.

 © 2017 The Authors

Figure EV4. RPA stimulation of XPF-ERCC1 activity on "+leading-strand" structure is not attributed to the displacement of the model nascent leading strand or the unwinding of the fork substrates by RPA.

A Nuclease activity of XE on the indicated fork substrates in the presence or absence of RPA. RPA specifically stimulates XE activity on a "simple fork" and "+leading-strand" substrates.

B Fluorescence anisotropy assay to determine the binding constants of RPA for either "simple fork" or "+lagging-strand" substrates. The blue diamonds denote the fluorophore-labelled nucleotides. Error bars represent SD, $n = 3$.

C (Top panel) Outline of potential consequences of incubating "+leading-strand" substrate radiolabelled on the model nascent leading strand with RPA, and the potential products that might be revealed by analysis on a non-denaturing PAGE gel. (Bottom panel) Nuclease activity performed as in panel a. Reaction products were separated on a 10% non-denaturing PAGE gel. The DNA substrates remain intact in the RPA alone reactions (lanes 4 and 9), indicating that RPA does not displace the model nascent leading strand or unwind the fork substrates, at the concentrations employed.

Source data are available online for this figure.

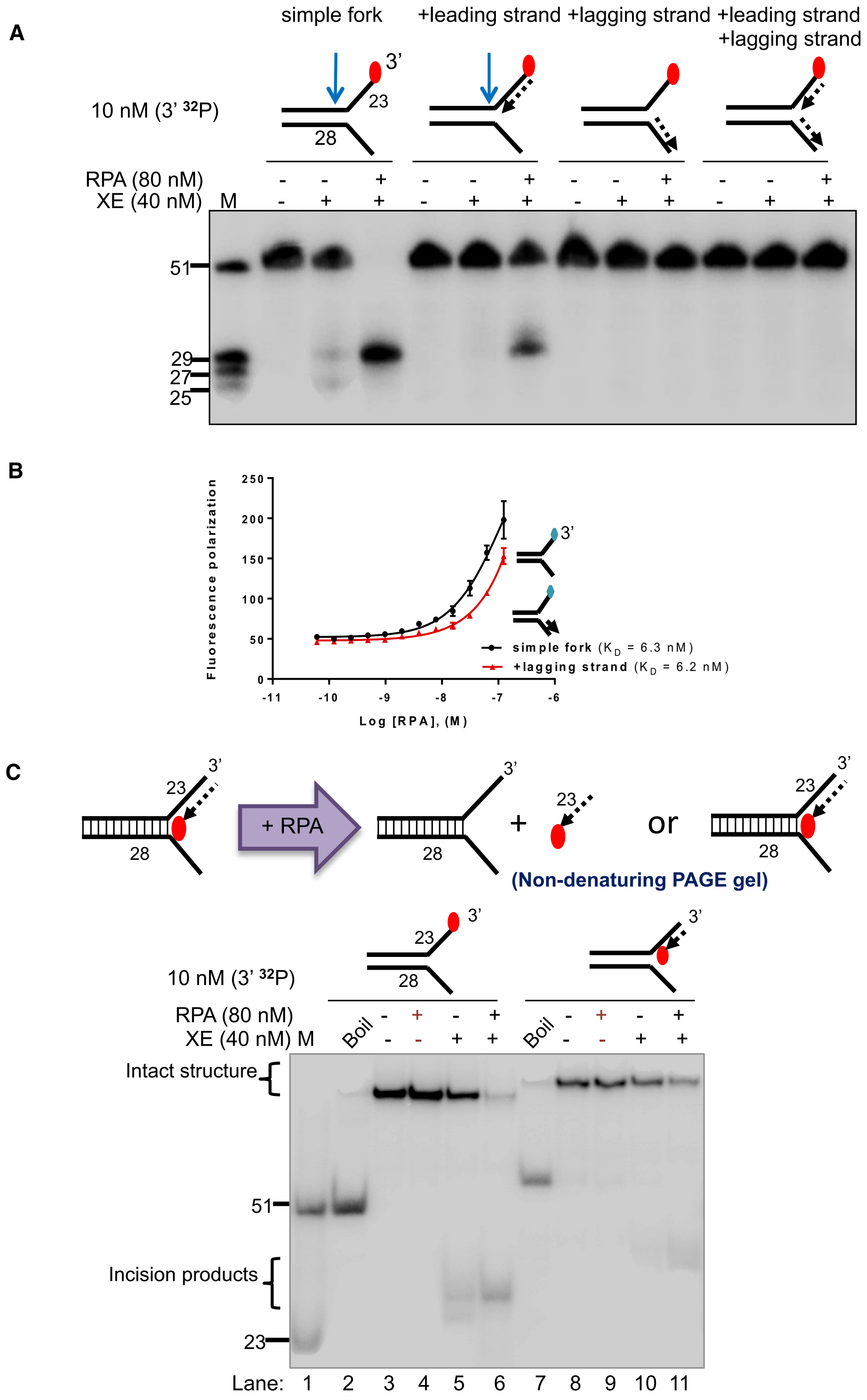

Figure EV4.

    © 2017 The Authors

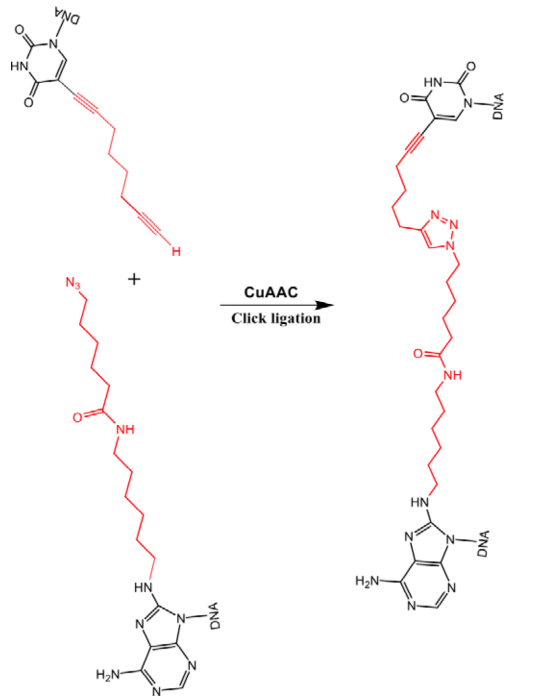

Figure EV5. Generation of triazole-containing crosslinked substrates.
Scheme of copper-catalysed click ligation between alkyne and azide oligonucleotides to form DNA interstrand crosslink (ICL) substrate.
