# Supplementary figures and images for: RPA activates the XPF‐ERCC1 endonuclease to initiate processing of DNA interstrand crosslinks
Source: EMBO J. 2017 Jun 12;36(14):2047–60. doi: 10.15252/embj.201796664 (PMC5510000; doi:10.15252/embj.201796664)

**A**

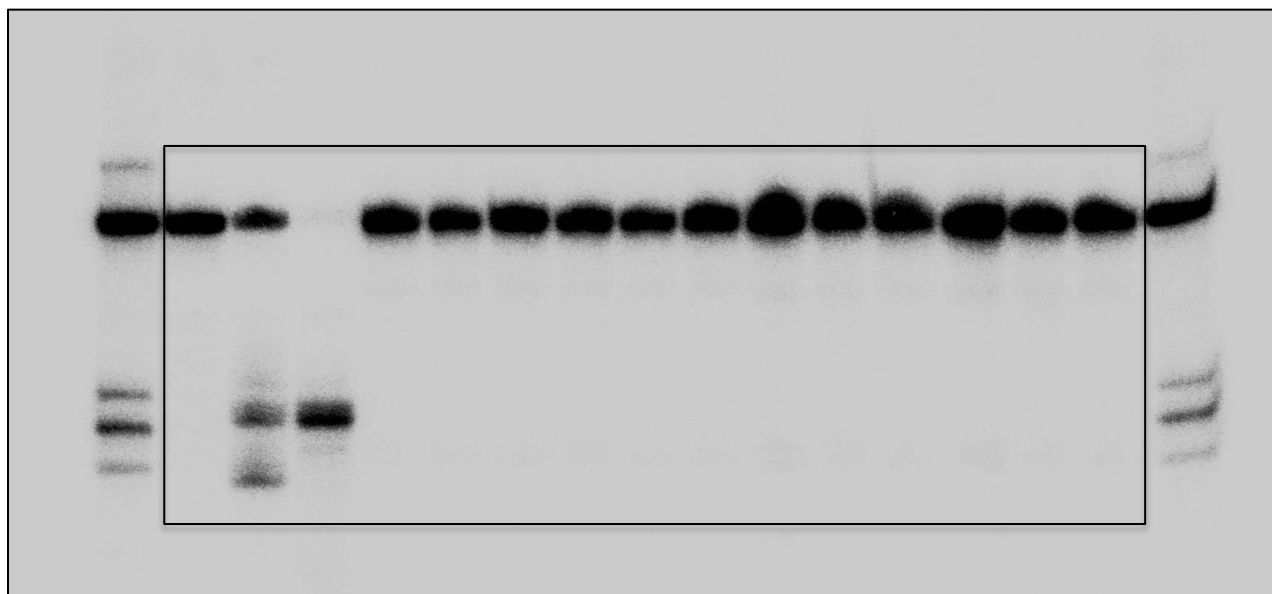

**B**

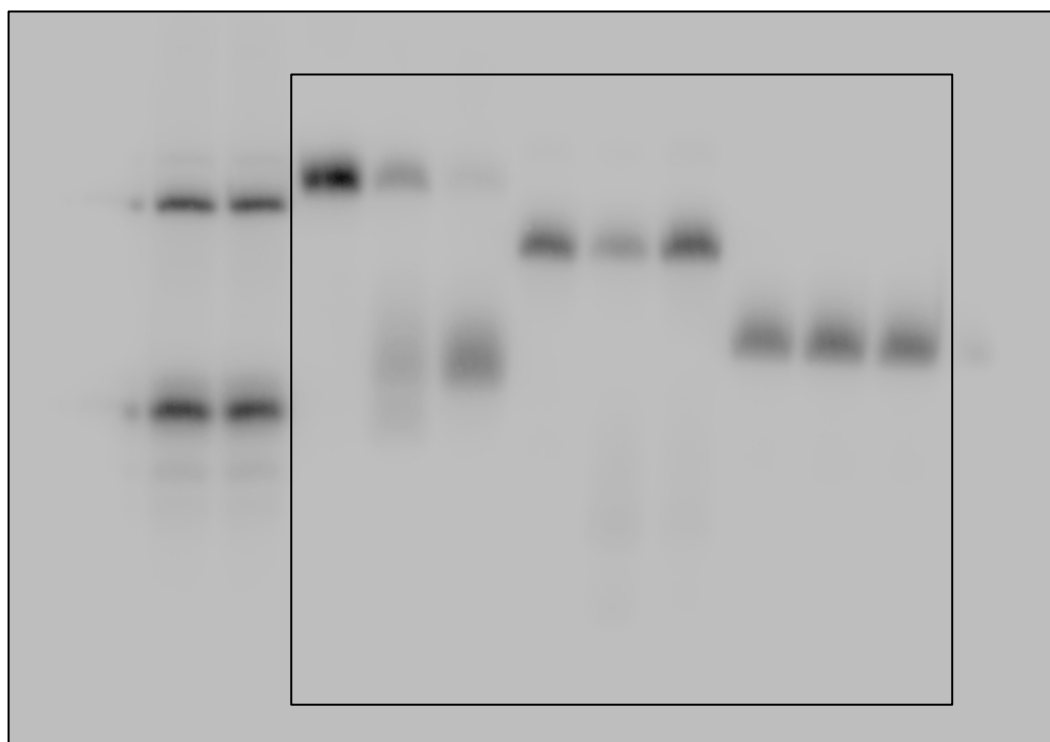

Supplement: Supplementary file 3 — Source Data for Expanded View [file EMBJ-36-2047-s003.zip › Abdullah_SD_EV3.pdf]

**A**

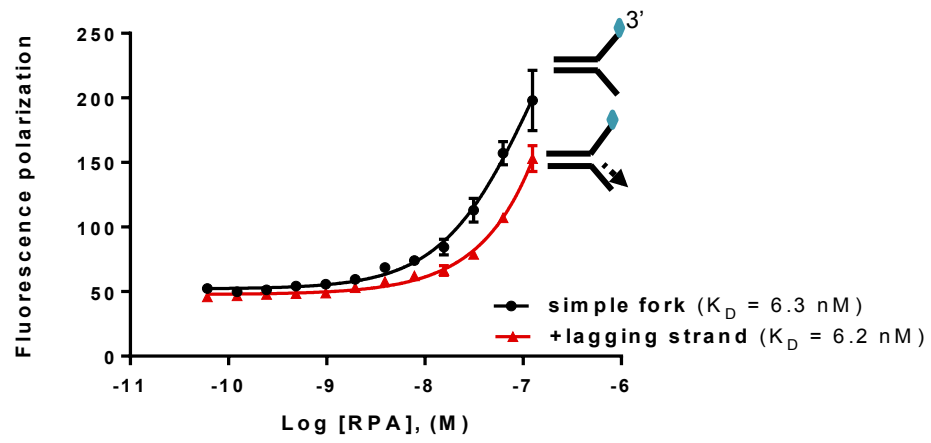

**B**

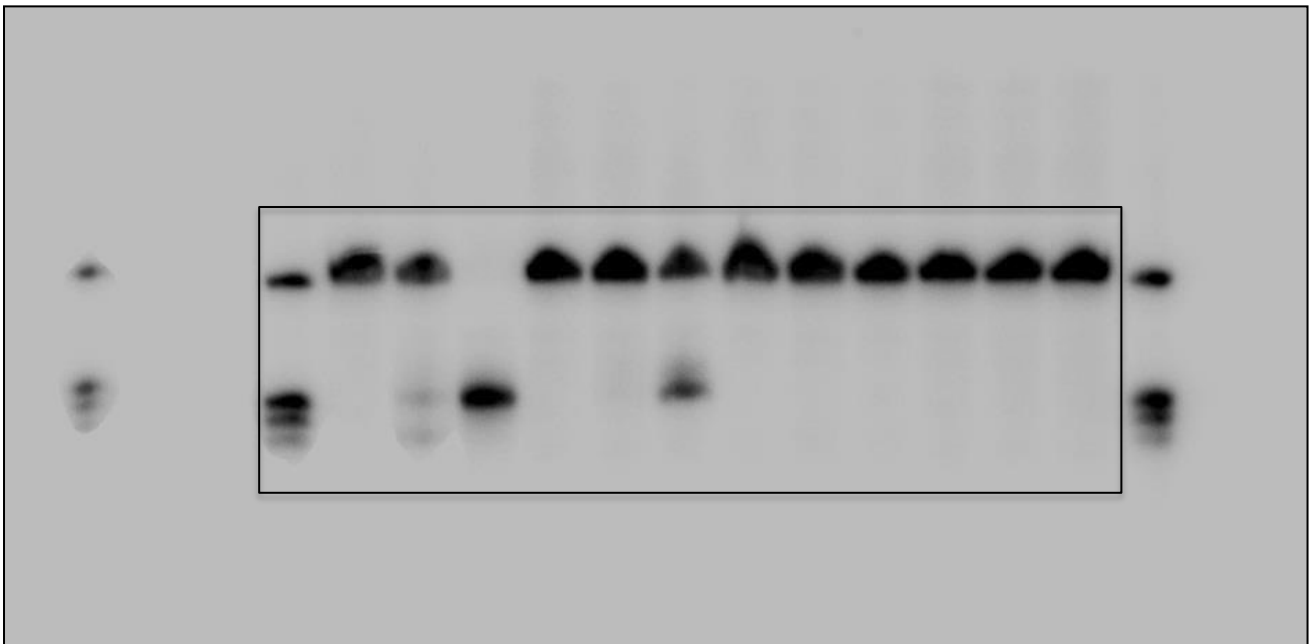

**C**

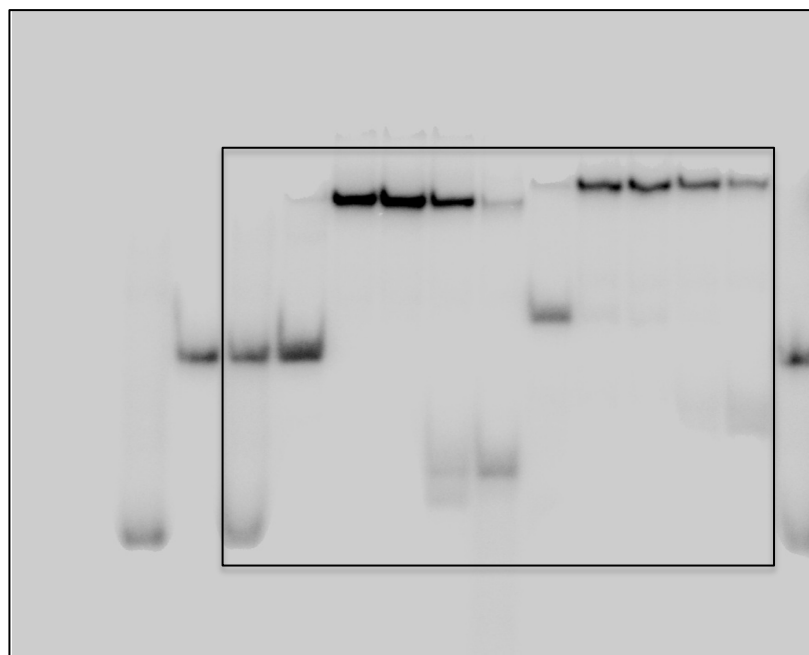

Supplement: Supplementary file 3 — Source Data for Expanded View [file EMBJ-36-2047-s003.zip › Abdullah_SD_EV4.pdf]
